# In vitro activity of anti-malarial ozonides against an artemisinin-resistant isolate

**DOI:** 10.1186/s12936-017-1696-0

**Published:** 2017-01-25

**Authors:** Fabian Baumgärtner, Joëlle Jourdan, Christian Scheurer, Benjamin Blasco, Brice Campo, Pascal Mäser, Sergio Wittlin

**Affiliations:** 1 Swiss Tropical and Public Health Institute, Socinstrasse 57, 4002 Basel, Switzerland; 2 University of Basel, Petersplatz 1, 4001 Basel, Switzerland; 3 Medicines for Malaria Venture, ICC, 20 Route de Pré-Bois, PO Box 1826, 1215 Geneva, Switzerland

**Keywords:** Ring-stage survival assay, Artemisinin, Ozonide, *Plasmodium falciparum*, Cam3.I^R539T^, Drug resistance

## Abstract

**Background:**

Recently published data suggest that artemisinin derivatives and synthetic peroxides, such as the ozonides OZ277 and OZ439, have a similar mode of action. Here the cross-resistance of OZ277 and OZ439 and four additional next-generation ozonides was probed against the artemisinin-resistant clinical isolate *Plasmodium falciparum* Cam3.I, which carries the K13-propeller mutation R539T (Cam3.I^R539T^).

**Methods:**

The previously described in vitro ring-stage survival assay (RSA_0–3h_) was employed and a simplified variation of the original protocol was developed.

**Results:**

At the pharmacologically relevant concentration of 700 nM, all six ozonides were highly effective against the dihydroartemisinin-resistant *P. falciparum* Cam3.I^R539T^ parasites, showing a per cent survival ranging from <0.01 to 1.83%. A simplified version of the original RSA_0–3h_ method was developed and gave similar results, thus providing a practical drug discovery tool for further optimization of next-generation anti-malarial peroxides.

**Conclusion:**

The absence of in vitro cross-resistance against the artemisinin-resistant clinical isolate Cam3.I^R539T^ suggests that ozonides could be effective against artemisinin-resistant *P. falciparum.* How this will translate to the human situation in clinical settings remains to be investigated.

**Electronic supplementary material:**

The online version of this article (doi:10.1186/s12936-017-1696-0) contains supplementary material, which is available to authorized users.

## Background

**Fig. 1:**
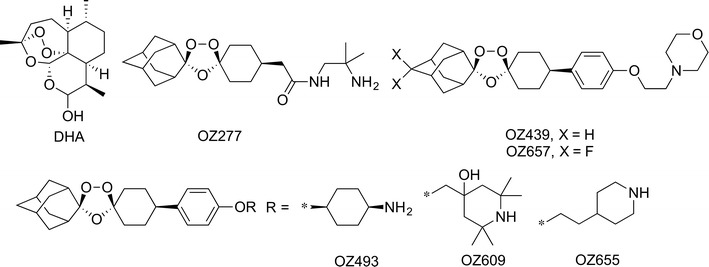
Chemical structures of dihydroartemisinin (DHA) and the six ozonides investigated

Malaria is one of the most important tropical diseases resulting in 214 million new cases and an estimated 438,000 malaria deaths worldwide in 2015 [1]. The discovery of artemisinin in the 1970s was an important step forward in anti-malarial drug therapy and was recognized with the Nobel Prize in Physiology or Medicine in 2015 [2, 3]. Artemisinin and its semi-synthetic derivatives, such as dihydroartemisinin (DHA) (Fig. 1), artesunate and artemether, contain a unique sesquiterpene lactone peroxide (1,2,4-trioxane) structure and artemisinin-based combination therapy (ACT) represents the current first-line treatment of uncomplicated *Plasmodium falciparum* malaria [4–6]. Since the starting material artemisinin is a natural product, its production is limited to the availability of the plant [4, 7], although several total syntheses of artemisinin have been described [8]. In 2004, Vennerstrom et al. reported the discovery of a completely synthetic peroxide anti-malarial containing a 1,2,4-trioxolane (ozonide) pharmacophore named OZ277 (arterolane) (Fig. 1) with anti-malarial activity comparable to the artemisinin derivatives [9, 10]. In combination with piperaquine, arterolane was registered for anti-malarial combination therapy in India in 2011 [11–14]. The next-generation ozonide, OZ439 (artefenomel) (Fig. 1), exhibits an increased pharmacokinetic half-life and good safety profile and is now being tested in phase IIb clinical trials [12, 14–17].

The iron-dependent alkylation hypothesis is one of the proposed modes of action of artemisinin and synthetic peroxides [18–21] where the peroxide is thought to be activated by the reductive cleavage in the presence of ferrous haem (or free Fe(II) derived from haem) released as a by-product of haemoglobin digestion in the food vacuole [20, 22–27]. Thereby carbon-centred radicals are generated, which then alkylate haem and parasite proteins [28–33]. The interaction of the artemisinin derivatives or ozonides with parasite targets is irreversible [31, 34]. Although the semi-synthetic artemisinins are highly effective, prolonged parasite clearance times were first reported along the Thai–Cambodian border in 2006, suggesting an emerging artemisinin resistance phenotype [35]. Today, delayed parasite clearance following treatment with artemisinin derivatives has been observed across Southeast Asia [36–41]. It was found that mutations in the Kelch 13 propeller domain are associated with ring-stage parasites entering a quiescent state with delayed parasite clearance after exposure to artemisinins [41–45]. When 50% inhibitory concentrations (IC_50_) were measured using conventional methods such as the [^3^H] hypoxanthine incorporation assay [46], no difference was observed between artemisinin-resistant and -susceptible strains after treatment with artemisinin or its derivatives [47–50]. In an effort to correlate the delayed parasite clearance observed in vivo with in vitro parasite survival, Witkowski et al. [48, 49] developed a ring-stage survival assay (RSA_0–3h_) that exploited the differences in susceptibility observed between wild-type and K13 mutants at the early ring stage of the asexual blood cycle following a short pulse of artemisinin treatment. In the RSA_0–3h_, synchronized young ring stage parasites (0–3 h old) are exposed to drugs for 6 h, and then cultured in drug free culture medium for 66 h before relative growth is determined by microscopic analysis [48, 49]. Since the structural analogies between artemisinins and ozonides (Fig. 1) suggest that they share similar modes of action, and thus some level of cross resistance [9, 10, 51, 52], the per cent survival of an artemisinin-resistant clinical isolate (Cam3.I^R539T^) treated with DHA, OZ277, OZ439, and four additional next-generation ozonides (Fig. 1) using the RSA_0–3h_ as described by Witkowski et al. [48, 49] was evaluated. Additionally, a sub-set of these compounds was tested in the RSA_0–3h_ described by Xie et al. [53] that also uses tightly synchronized ring-stage cultures, but allows the assay to be performed routinely within a convenient time-frame.

## Methods

### Parasite cultivation

The artemisinin-resistant *P. falciparum* isolate Cam3.I^R539T^ from Battambang, Cambodia was obtained from BEI Resources [54] with the accession number MRA-1240. The drug-sensitive *P. falciparum* strain NF54 (airport strain from The Netherlands) was provided by F. Hoffmann-La Roche Ltd. Parasites were cultivated in standard cultivation medium, consisting of hypoxanthine (50 mg/l), RPMI (10.44 g/l) supplemented with HEPES (5.94 g/l), albumax (5 g/l), sodium bicarbonate (2.1 g/l) and neomycin (100 mg/l) [55].

### Ring-stage survival assays (RSA_0–3h_)

Ring-stage survival assays (RSA_0–3h_) were carried out essentially as previously described by Witkowski et al. [48], but with a few modifications in the drug-washing procedure to ensure that no residual peroxide was present during the 66-h post-treatment period [56]. Briefly, zero to 3 h post-invasion ring stages were adjusted to 1% parasitaemia and 2.5% haematocrit by adding uninfected erythrocytes, transferred in a total volume of 1 ml into 48-well plates and exposed for 6 h to a range of concentrations (700, 350, 175, 88, and 49 nM) of DHA or one of the six ozonides tested in this study. The synthesis of the four next-generation ozonides, OZ493, OZ609, OZ655 and OZ657, will be reported in due course by the laboratory of Prof. Jonathan Vennerstrom (pers. comm.). After 6 h, cultures were transferred to 15 ml conical tubes, centrifuged at 1400 rpm (400*g*) for 2 min and carefully washed two times with 12 ml of culture medium. The complete removal of compound after washing was verified by incubating the supernatant recovered after the last washing step with fresh cultures of NF54 parasites, ensuring that no growth inhibition was detected. After washing, blood pellets were resuspended in complete drug-free culture medium, transferred into new wells and cultured for 66 h under standard conditions.

Thin blood smears were prepared, methanol-fixed and stained with 10% Giemsa. Per cent survival was assessed using light microscopy, counting the number of parasitized cells in ≥10,000 red blood cells (RBCs) and comparing survival to that of the drug-free dimethylsulfoxide incubation. Microscopy analysis was performed independently by two microscopists, one having more than 15 years of work experience.

### Alternative parasite synchronization method

Parasites were synchronized according to Xie et al. [53] with 5% D-sorbitol. After 30 and 43 h, parasites were synchronized a second and third time, respectively, resulting in zero to 1-h old ring-stage parasites. The RSA_0–3h_ was initiated 2 h later.

### Standard [^3^H] hypoxanthine incorporation assay

The in vitro anti-malarial activity was measured using the [^3^H]-hypoxanthine incorporation assay [55]. Results were expressed as the concentration resulting in 50% inhibition (IC_50_).

## Results

**Fig. 2:**
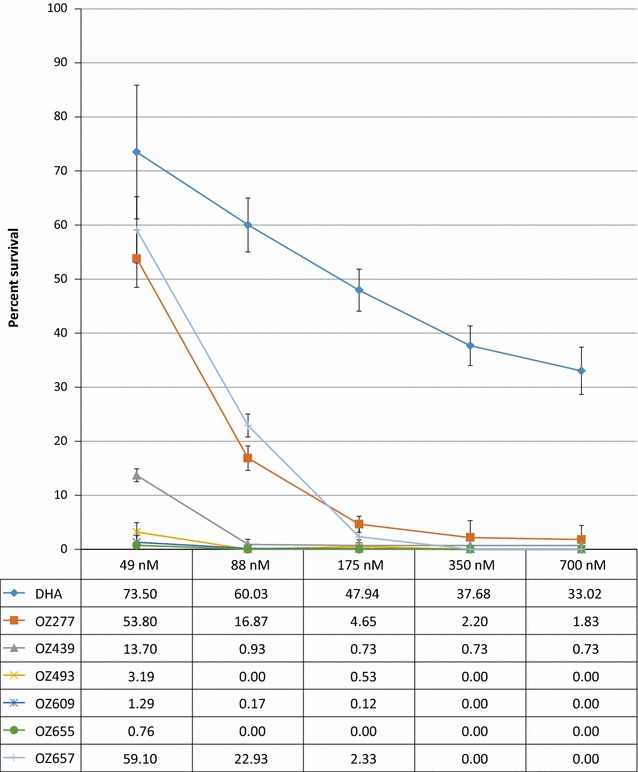
Mean per cent survival ± standard error (SE) of *Plasmodium falciparum* isolate Cam3.I^R539T^ parasites after a 6-h exposure to a range of concentrations of dihydroartemisinin (DHA) or six different ozonides. Three biological replicates were performed per compound

The per cent survival of parasites exposed to a concentration range of DHA and six different ozonides (Fig. 1) was determined using the artemisinin-resistant *P. falciparum* Cambodian isolate Cam3.I^R539T^. As expected, DHA exposure gave a high survival rate ranging from 74 to 33% at concentrations of 49 and 700 nM, respectively (Fig. 2), which is comparable to the observed survival value of 40% at 700 nM published previously [44]. In contrast, when tested at 700 nM, the two ozonides OZ277 and OZ439 showed an approximate 18- to 45-fold increase in potency compared with DHA (Fig. 2). Full and equal potency was observed when DHA, OZ277 and OZ439 were tested in parallel in the RSA_0–3h_ using the artemisinin-sensitive strain NF54 (Additional file 1: Table S1). At the lowest concentration (49 nM), OZ277 had poor activity, showing a similar per cent survival to that of DHA, whereas OZ439 was still about fivefold more potent. A possible explanation for OZ439 being more potent than OZ277 could be related to its improved stability in blood as previously described [15]. In those studies, OZ277 or OZ439 were incubated at 37 °C in *P. falciparum*-infected human blood. After 2 h more than 90% of OZ277 was degraded, whereas OZ439 was found to be about 10–20× more stable. A similar and more recent study found similar differences in stability for OZ277 and OZ439 [56]. The same compounds were also tested in a more convenient variation of the standard RSA_0–3h_ that uses synchronized ring-stage cultures that can be easily produced during normal working hours [53]. As shown in Table 1, this alternative synchronization method gave results that were comparable to those obtained using the standard RSA_0–3h_.

**Table 1:** Mean per cent survival (individual values in brackets) of Cam3.I^R539T^ isolate after 6 h exposure to a range of concentrations of DHA, OZ439 or OZ277 using the synchronization protocol from Xie et al. [53]

Compounds	RSA values (% survival) at different concentrations		
	175 nM	350 nM	700 nM
DHA	46 (49, 43)	42 (45, 39)	37 (39, 35)
OZ277	4.0 (4.4, 3.6)	2.3 (1.9, 2.7)	1.4 (1.7, 1.1)
OZ439	<0.01 (<0.01, <0.01)	<0.01 (<0.01, <0.01)	<0.01 (<0.01, <0.01)

Two biological replicates were performed per compound

To investigate further the level of cross-resistance between DHA and the ozonides, four additional next-generation ozonides (OZ493, OZ609, OZ655, OZ657) (Fig. 1) were tested against the Cam3.I^R539T^ parasites. While all six ozonides had a similar IC_50_ value using a conventional 72-h [^3^H] hypoxanthine incorporation assay (Additional file 1: Table S2), the RSA_0–3h_ showed that OZ493, OZ609 and OZ655 were highly potent and completely inhibited the growth of the artemisinin-resistant isolate at the two highest concentrations tested (Fig. 2). At the lowest concentration, potency was comparable to that for OZ439. The overall potency of OZ657 was comparable to that of OZ277.

The RSA_0–3h_ was recently developed to provide an in vitro correlate of the longer in vivo parasite clearance times observed after artemisinin treatment in Southeast Asia, which is widely interpreted as a sign of potential artemisinin resistance [57, 58]. Provided that the RSA_0–3h_ does indeed predict the potency of compounds against artemisinin-resistant parasites in malaria patients, the here described data suggest that all of the tested ozonides are highly potent against isolates such as *P. falciparum* Cam3.I^R539T^. These data are in line with the recent clinical observation that the parasite clearance rate following OZ439 treatment is not significantly affected by resistance-associated mutations in the Kelch 13 propeller region [17] and the recent data published by Siriwardana et al. [59], which showed no reduced susceptibility of OZ439 in a different delayed clearance phenotype parasite (Cam3.II) in vitro.

## Conclusion

In the traditional RSA_0–3h_, as well as a more convenient variation of the original method, all of the tested ozonides, were highly potent against the artemisinin-resistant isolate *P. falciparum* Cam3.I^R539T^ in contrast to results for DHA. These data indicate that artemisinin-resistant *P. falciparum* infections could be successfully treated with ozonide anti-malarial drugs.

## Additional files

**Additional file 1: Table S1.** Mean per cent survival (individual values in brackets) of NF54 after 6 h exposure to 500 nM of DHA, OZ439 or OZ277 using the synchronization protocol from Straimer et al. [44]. **Table S2**. Mean in vitro IC50 values (single values in brackets) for *Plasmodium falciparum* isolate Cam3.IR539T and NF54 in the 72-h [^3^H] hypoxanthine assay.

## Abbreviations

OZ277: arterolane
OZ439: artefenomel
DHA: dihydroartemisinin
ACT: artemisinin combination therapy
RBC: red blood cell
