# Supplementary material for: In vitro activity of anti-malarial ozonides against an artemisinin-resistant isolate
Source: Malar J. 2017 Jan 25;16:45. doi: 10.1186/s12936-017-1696-0 (PMC5267415; doi:10.1186/s12936-017-1696-0)
Supplement: Supplementary file 1 — Additional file 1: Table S1. Mean per cent survival (individual values in brackets) of NF54 after 6 h exposure to 500 nM of DHA, OZ439 or OZ277 using the synchronization protocol from Straimer et al. [44]. Table S2. Mean in vitro IC50 values (single values in brackets) for Plasmodium falciparum isolate Cam3.IR539T and NF54 in the 72-h [3H] hypoxanthine assay. [file 12936_2017_1696_MOESM1_ESM.docx]

**Supplementary Table S1 Mean per cent survival (individual values in brackets) of NF54 after 6 hours exposure to 500 nM of DHA, OZ439 or OZ277 using the synchronization protocol from Straimer *et al*. [44.]**

Two biological replicates were performed per compound.

| Compounds | Per cent survival | | |
| --- | --- | --- | --- |
| DHA | | <0.01 (<0.01,<0.01) |  |
| OZ277 | | <0.01 (<0.01,<0.01) |  |
| OZ439 | | <0.01 (<0.01,<0.01) |  |

**Supplementary Table S2 Mean *in vitro* IC_50_ values (single values in brackets) for *Plasmodium falciparum* isolate Cam3.I^R539T^ and NF54 in the 72-hour [^3^H] hypoxanthine assay**

Two biological replicates were performed per compound.

| Compounds | IC_50_ (nM)  Cam3.I^R539T^ | | | IC_50_ (nM)  NF54 | |
| --- | --- | --- | --- | --- | --- |
| DHA | | 1.5 (1.7,1.3) | 2.6 (2.4,2.8) | |  |
| OZ277 | | 1.8 (1.8,1.8) | 1.7 (1.7,1.8) | |  |
| OZ439 | | 4.6 (4.6,4.6) | 4.2 (4.6,3.9) | |  |
| OZ493 | | 3.6 (3.6,3.7) | 3.8 (4.5,3.1) | |  |
| OZ609 | | 1.8 (1.8,1.8) | 1.3 (1.4,1.2) | |  |
| OZ655 | | 2.8 (2.7,2.8) | 2.5 (2.3,2.7) | |  |
| OZ657 | | 2.9 (2.9,2.9) | 2.3 (2.3,2.3) | |  |
